# Supplementary material for: Effects of vitamin D on COVID-19 risk and hospitalisation in the UK biobank
Source: PLoS One. 2025 Jul 18;20(7):e0328232. doi: 10.1371/journal.pone.0328232 (PMC12273939; doi:10.1371/journal.pone.0328232)
Supplement: S1 Table — (DOCX) [file pone.0328232.s001.docx]

**S1 table. Ethnicities included in UK Biobank.**

| **Combined ethnicities** | **UK Biobank ethnicities** |
| --- | --- |
| White | White British  White Irish  Any other white background |
| Mixed | White and Black Caribbean  White and Black African  White and Asian  Any other mixed background |
| Asian | Indian  Pakistani  Bangladeshi  Chinese  Any other Asian background |
| Black | Caribbean  African  Any other Black background |
| Other | Other ethnic group |
