# Supplementary material for: Effects of vitamin D on COVID-19 risk and hospitalisation in the UK biobank
Source: PLoS One. 2025 Jul 18;20(7):e0328232. doi: 10.1371/journal.pone.0328232 (PMC12273939; doi:10.1371/journal.pone.0328232)
Supplement: S2 Table — *Model A- Adjusted for Sex, Age at recruitment, Townsend Deprivation Index, overall health rating, BMI, and smoking status, with normal Vitamin D status as reference. (DOCX) [file pone.0328232.s002.docx]

**S2 table. Stratified analyses for COVID-19 infection within the total population.**

|  | Vitamin D status* | | | | | |
| --- | --- | --- | --- | --- | --- | --- |
|  | Insufficient | | | Deficient | | |
|  | OR | 95%CI | p-value | OR | 95%CI | p-value |
| White | 0.97 | 0.86-0.95 | <0.00 | 0.90 | 0.86-0.95 | 0.12 |
| Mixed | 1.08 | 0.71-1.64 | 0.70 | 1.02 | 0.61-1.68 | 0.93 |
| Asian | 0.97 | 0.69-1.35 | 0.86 | 1.50 | 1.08-2.07 | 0.01 |
| Black | 1.29 | 0.95-1.76 | 0.09 | 1.57 | 1.14-2.16 | 0.005 |
| Other | 1.08 | 0.73-1.59 | 0.66 | 0.97 | 0.64-1.48 | 0.92 |

*Model A- Adjusted for Sex, Age at recruitment, Townsend Deprivation Index, overall health rating, BMI, and smoking status, with normal Vitamin D status as reference.
