# Supplementary material for: Effects of vitamin D on COVID-19 risk and hospitalisation in the UK biobank
Source: PLoS One. 2025 Jul 18;20(7):e0328232. doi: 10.1371/journal.pone.0328232 (PMC12273939; doi:10.1371/journal.pone.0328232)
Supplement: S3 Table — *Model A- Adjusted for Sex, Age at recruitment, Townsend Deprivation Index, overall health rating, BMI, and smoking status, with normal Vitamin D status as reference. (DOCX) [file pone.0328232.s003.docx]

**S3 table. Stratified analyses for COVID-19 hospitalisation within the total population.**

|  | Vitamin D status* | | | | | |
| --- | --- | --- | --- | --- | --- | --- |
|  | Insufficient | | | Deficient | | |
|  | OR | 95%CI | p-value | OR | 95%CI | p-value |
| White | 1.19 | 1.08-1.32 | <0.00 | 1.44 | 1.25-1.66 | <0.00 |
| Mixed | 0.44 | 0.12-1.54 | 0.20 | 0.78 | 0.20-2.96 | 0.72 |
| Asian | 0.90 | 0.38-2.09 | 0.80 | 0.59 | 0.26-1.35 | 0.21 |
| Black | 0.95 | 0.53-3.04 | 0.58 | 1.27 | 0.53-3.04 | 0.58 |
| Other | 1.86 | 0.57-5.99 | 0.29 | 1.54 | 0.43-5.44 | 0.49 |

*Model A- Adjusted for Sex, Age at recruitment, Townsend Deprivation Index, overall health rating, BMI, and smoking status, with normal Vitamin D status as reference.
