# Supplementary material for: Effects of vitamin D on COVID-19 risk and hospitalisation in the UK biobank
Source: PLoS One. 2025 Jul 18;20(7):e0328232. doi: 10.1371/journal.pone.0328232 (PMC12273939; doi:10.1371/journal.pone.0328232)
Supplement: S4 Table — *Model A- Adjusted for Sex, Age at recruitment, Townsend Deprivation Index, overall health rating, BMI, and smoking status, with normal Vitamin D status as reference. (DOCX) [file pone.0328232.s004.docx]

**S4 table. Stratified analyses for COVID-19 infection within the cancer population.**

|  | Vitamin D status* | | | | | |
| --- | --- | --- | --- | --- | --- | --- |
|  | Insufficient | | | Deficient | | |
|  | OR | 95%CI | p-value | OR | 95%CI | p-value |
| White | 1.02 | 0.93-1.12 | 0.59 | 0.94 | 0.81-1.08 | 0.42 |
| Mixed | 1.08 | 0.26-4.41 | 0.90 | 1.14 | 0.23-5.47 | 0.86 |
| Asian | 1.41 | 0.34-5.81 | 0.62 | 1.27 | 0.30-5.29 | 0.73 |
| Black | 1.17 | 0.42-3.24 | 0.75 | 3.50 | 1.22-10.01 | 0.01 |
| Other | 1.17 | 0.24-5.57 | 0.83 | 2.27 | 0.49-10.56 | 0.29 |

*Model A- Adjusted for Sex, Age at recruitment, Townsend Deprivation Index, overall health rating, BMI, and smoking status, with normal Vitamin D status as reference.
