# Supplementary material for: Effects of vitamin D on COVID-19 risk and hospitalisation in the UK biobank
Source: PLoS One. 2025 Jul 18;20(7):e0328232. doi: 10.1371/journal.pone.0328232 (PMC12273939; doi:10.1371/journal.pone.0328232)
Supplement: S5 Table — *Model A- Adjusted for Sex, Age at recruitment, Townsend Deprivation Index, overall health rating, BMI, and smoking status, with normal Vitamin D status as reference. (DOCX) [file pone.0328232.s005.docx]

**S5 table. Stratified analyses for COVID-19 hospitalisation within the cancer population.**

|  | Vitamin D status* | | | | | |
| --- | --- | --- | --- | --- | --- | --- |
|  | Insufficient | | | Deficient | | |
|  | OR | 95%CI | p-value | OR | 95%CI | p-value |
| White | 1.21 | 0.94-1.55 | 0.12 | 1.37 | 0.94-2.01 | 0.09 |
| Mixed | - | - | - | - | - | - |
| Asian | 1.24 | 0.06-25.65 | 0.88 | 1.19 | 0.04-34.28 | 0.91 |
| Black | 0.55 | 0.02-10.46 | 0.69 | 2.02 | 0.10-39.10 | 0.63 |
| Other | - | - | - | - | - | - |

*Model A- Adjusted for Sex, Age at recruitment, Townsend Deprivation Index, overall health rating, BMI, and smoking status, with normal Vitamin D status as reference.
