# Supplementary material for: Effects of vitamin D on COVID-19 risk and hospitalisation in the UK biobank
Source: PLoS One. 2025 Jul 18;20(7):e0328232. doi: 10.1371/journal.pone.0328232 (PMC12273939; doi:10.1371/journal.pone.0328232)
Supplement: Attachment — Submitted filename: Response to reviewers.docx [file pone.0328232.s008.docx]

**Response to reviewers**

**AUTHORS’ REPLY: Thank you very much for your incredibly useful references and insightful comments. We have updated the outdated references with those you kindly provided and included the study on delays between symptom onset and hospital admission into our discussion.**

**In particular, we have revised the following paragraph of the discussion to incorporate these points more clearly:**

*“Although epidemiological evidence on the link between VitD and COVID-19 severity remains inconsistent, there are strong biological reasons to support a potential connection. VitD plays a crucial regulatory role in both acquired and innate immunity, and higher levels have been associated with a reduced risk of the cytokine storm and subsequent acute respiratory distress syndrome (ARDS), a leading cause of COVID-19 mortality (34, 35). Two key mechanisms by which VitD may exert protective effects include the upregulation of antimicrobial peptides such as cathelicidin (LL-37), which reduce viral and bacterial viability, and the suppression of pro-inflammatory cytokines that contribute to disease progression. These effects are likely most beneficial early in the course of infection (36). Importantly, clinical studies have highlighted the complexity of COVID-19 progression, including evidence that shows delays between symptom onset and hospital admission which may in turn influence patient outcomes (37, 38). Furthermore, acute inflammation itself may reduce circulating 25(OH)D concentrations, complicating the interpretation of VitD measurements taken during or shortly after illness onset (39, 40). In our study, no significant association was observed between VitD levels and COVID-19 infection or hospitalization among participants with a prior cancer diagnosis. This lack of association may be due to immune suppression from cancer treatments, such as chemotherapy, which could blunt the immune-modulating effects of VitD (41). Alternatively, the chronic inflammation and immune dysfunction commonly seen in cancer patients might overshadow any potential protective benefits of adequate VitD levels (42). Further studies are needed to disentangle the interplay between cancer, immune function, and VitD in the context of COVID-19.”*
